# Closed-form solution for a cantilevered sectorial plate subjected to a tip concentrated force

**DOI:** 10.1186/s40064-016-2473-1

**Published:** 2016-06-21

**Authors:** Carl W. Christy, David C. Weggel, R. E. Smelser

**Affiliations:** Zapata Incorporated, 6302 Fairview Road, Suite 600, Charlotte, NC 28210 USA; Department of Civil and Environmental Engineering, UNC Charlotte, 9201 University City Blvd., Charlotte, NC 28223 USA; The William States Lee College of Engineering, UNC Charlotte, 9201 University City Blvd., Charlotte, NC 28223 USA

**Keywords:** Sectorial plate, Cantilever, Concentrated force, Bending moment, Twisting moment

## Abstract

A closed-form solution is presented for a cantilevered sectorial plate subjected to a tip concentrated force. Since the particular solution for this problem was not found in the literature, it is derived here. Deflections from the total solution (particular plus homogeneous solutions) are compared to those from a finite element analysis and are found to be in excellent agreement, producing an error within approximately 0.08 %. Normalized closed-form deflections and slopes at the fixed support, resulting from an approximate enforcement of the boundary conditions there, deviate from zero by <0.08 %. Finally, the total closed-form solutions for a cantilevered sectorial plate subjected to independent applications of a tip concentrated force, a tip bending moment, and a tip twisting moment, are compiled.

## Background

Solutions to sectorial plate problems have been investigated since the early twentieth century. Today, besides being of academic interest, these solutions are useful for verifying finite element analysis results, performing parametric studies, or assisting with preliminary designs. Timoshenko and Woinowsky-Krieger (1959) presented solutions for sectorial plates fixed (clamped) along the circular boundary and simply supported along the (straight) radial edges. However, they acknowledged that sectorial plate solutions containing clamped or free radial edges must be solved using approximate methods. Williams (1952a, b) presented solutions showing the stress singularities that develop due to various boundary conditions for plates in bending and extension; these solutions were developed using eigenfunction expansions.

Barber (1979) investigated the deflections of annular sectorial plates for some problems with concentrated moments and forces on a plate with a straight edge. He also provided a solution for twisting moments applied at the vertex of an infinite sectorial plate. Lim and Wang (2000) developed solutions for annular Mindlin sectorial plates using the Kirchhoff solutions, and Boonchareon et al. (2013) revisited the William’s problem for plate bending, providing solutions for problems that were previously unsolved. Huang et al. (2016) solved the infinite sectorial plate problem subjected to tip loads consisting of two twisting moments and a bending moment in a functionally graded plate, as well as concentrated forces in the plane of the plate; these solutions employed complex variable techniques.

Nadai (1925) provided particular solutions to a sectorial plate subjected to a tip twisting moment and a tip bending moment. These solutions are applicable to a plate of infinite radial extent with free radial edges, implying that any additional boundary conditions are located at a far distance from the plate’s tip. Carrier and Shaw (1950) applied an asymmetric eigenfunction expansion to Nadai’s particular solution for the tip twisting moment problem to account for fixed circumferential boundary conditions for a cantilevered sectorial plate; they enforced the fixed boundary conditions in a relaxed (averaged) manner. Kennedy et al. (2008) corrected mistakes in the presentation of Carrier and Shaw and compared their results to those from a finite element analysis; the agreement was found to be very good. Similarly, Christy et al. (2013) used Nadai’s particular solution for a sectorial plate subjected to a tip bending moment and applied a symmetric eigenfunction expansion for the fixed boundary conditions, similar to the procedure used by Carrier and Shaw (1950), to produce a closed-form deflection solution.

This paper presents the total solution for a cantilevered sectorial plate subjected to a tip concentrated force. However, the particular solution for this problem was not found in the literature; so it is first derived and presented in this paper. Then the particular solution is modified by a symmetric eigenfunction expansion and an “averaged” application of the fixed boundary conditions to produce the closed-form deflection solution. The solution is subsequently compared to the results from a finite element analysis.

## Results and discussion

**Fig. 1:**
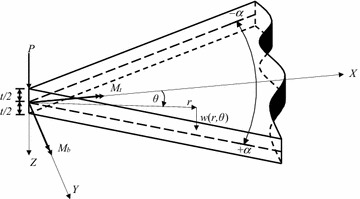
Infinite sectorial plate subject to three applied tip loading conditions

The infinite sectorial plate shown in Fig. 1 is subjected to three different applied tip loading conditions: a twisting moment *M*_*t*_, a bending moment *M*_*b*_, and a concentrated force *P*; the applied moments are observed to have the traditional units of force times length. The coordinate systems and the plate dimensions are also shown in the diagram. The origin of the Cartesian coordinate system is located at the tip of the plate on the plate’s neutral surface. A right-hand coordinate system is used, where the positive *X*-axis points to the right, bisecting the plate, the *Y*-axis is perpendicular to the *X*-axis, and the positive *Z*-axis points downward. The origin of the polar coordinate system is also located at the tip of the plate. The radial distance *r* ranges from 0 (at the tip) to infinity; if the plate were to have a finite length *R*, *r* would satisfy 0 ≤ *r* ≤ *R*. The circumferential coordinate *θ* measures in-plane angles and ranges over −*α* ≤ *θ* ≤ + *α*, where 2*α* is the angular extent of the plate. Positive tip moments are shown by double headed arrows that follow the right hand rule and point along the positive *X*- and *Y*-axes. The positive concentrated force *P* acts in the direction of the positive *Z*-axis.

### Existing particular solutions for tip twisting moment and tip bending moment

Nadai (1925) presented mixed coordinate solutions (i.e. a function of both Cartesian and polar coordinates) for an infinite sectorial plate subjected to a tip twisting moment and a tip bending moment. Equivalent equations, after being converted to polar coordinates, are presented below.

As presented in Kennedy et al. (2008), the particular solution for a sectorial plate subjected to a tip twisting moment is

1 $$w_{0t} \; = \;C_{t} \left( {r\ln \frac{r}{R}\sin \theta \; + \;\frac{1 + v}{2}r\theta \cos \theta } \right)$$

where

2 $$C_{t} \; = \;\frac{{ - 2M_{t} }}{{D[(1 - v)^{2} \sin (2\alpha ) + 2\alpha (1 - v)(3 + v)]}}$$

Similarly, Christy et al. (2013) presented the particular solution for a sectorial plate subjected to a tip bending moment

3 $$w_{0b} \; = \;C_{b} \left( {r\ln \frac{r}{R}\cos \theta \; - \;\frac{1 + v}{2}r\theta \sin \theta } \right)$$

where

4 $$C_{b} \; = \;\frac{{ - 2M_{b} }}{{D[(1 - v)^{2} \sin (2\alpha ) - 2\alpha (1 - v)(3 + v)]}}$$

In these equations *w*_0*i*_ is the plate’s deflection in the *Z*-direction, where the first subscript 0 denotes the particular solution and the second subscript, *t* or *b*, denotes that the deflection is due to an applied twisting moment or bending moment, respectively. *R* is an arbitrary radial length, *D* is the plate’s flexural rigidity (*D* = *Et*^3^/[12(1 − *ν*^2^)]), *ν* and *E* are the Poisson’s ratio and Young’s modulus of the material, respectively, and *t* is the plate’s thickness. The similarities of Eqs. (1) and (2) to Eqs. (3) and (4) are readily observed. However, Eqs. (1) and (2) represent an asymmetric solution with respect to the *X*-axis due to the applied twisting moment, and Eqs. (3) and (4) represent a symmetric solution due to the applied bending moment.

### Derivation of the particular solution for a tip concentrated force

To the authors’ knowledge, the particular solution for an infinite sectorial plate subjected to a tip concentrated force does not exist in the literature, so it is derived here. An infinite sectorial plate implies a self-similar problem, leading to a deflection solution of the form

5 $$w_{0} \; = r^{n} \cdot f(\theta )$$

Equilibrium requires that *n* = 2, since the shear force per length along any arc must be proportional to *r*^−1^. If *θ* is restricted to the range −*α* ≤ *θ* ≤ + *α*, the solution to the biharmonic equation gives the deflection solution

6 $$w_{0} \; = r^{2} \left( {B + C\cos (2\theta )} \right)$$

The general solution to the biharmonic equation is given in Timoshenko and Goodier (1987). The relationship between the two constants, *B* and *C*, is obtained by applying the free radial edge condition *M*_*θ*_(*r*, *α*) = 0, where

7 $$M_{\theta } \left( {r,\theta } \right) = - D\left[ {\frac{1}{r}\frac{\partial w(r,\theta )}{\partial r} + \frac{1}{{r^{2} }}\frac{{\partial^{2} w(r,\theta )}}{{\partial \theta^{2} }} + \nu \frac{{\partial^{2} w(r,\theta )}}{{\partial r^{2} }}} \right]$$

is generally an internal moment that induces normal stresses in the plate in the *θ* direction; *M*_*θ*_(*r*, *θ*) is a “moment intensity” with units of moment per length (N m/m). Thus, substituting Eq. (6) and its derivatives into Eq. (7), evaluated at *θ* = +*α*, gives the expression

8 $$B\; = \;\frac{C\cos (2\alpha )(1 - v)}{(1 + v)}$$

A second equation relating *P* and *C* is obtained by using the expression for the internal twisting moment (a moment intensity with N m/m units)

9 $$M_{r\theta } \left( {r,\theta } \right) = - D(1 - v)\left[ {\frac{1}{r}\frac{{\partial^{2} w(r,\theta )}}{\partial r\partial \theta } - \frac{1}{{r^{2} }}\frac{\partial w(r,\theta )}{\partial \theta }} \right]$$

When Eq. (6) and its derivatives are substituted into Eq. (9) and evaluated at *θ* = + *α*, it produces

10 $$M_{r\alpha } \left( {r,\alpha } \right) = 2(1 - v)DC\sin (2\alpha )$$

**Fig. 2:**
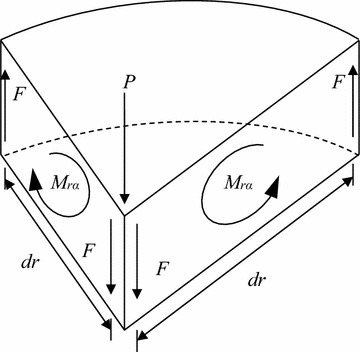
Corner forces near plate tip created by twisting moment

The twisting moment intensity along the plate’s edge is observed to remain constant for all values of *r*. As shown in Fig. 2, the twisting moment intensity *M*_*rα*_ times the length d*r* produces a moment in force times length units that can be replaced by equivalent couple forces *F* separated by the same distance d*r*; thus *F*d*r* = *M*_*rα*_d*r*, which leads to

11 $$F = M_{r\alpha }$$

The two corner forces *F* toward the tip of the plate, from two different twisting moment couples (see Fig. 2), are set equal the applied concentrated force *P* to give

12 $$P = 2F$$

Substituting Eq. (11) into Eq. (12) shows that

13 $$P = 2M_{r\alpha }$$

Next, substituting Eq. (10) into Eq. (13) and rearranging gives the expression for *C*

14 $$C = \frac{P}{4D(1 - v)\sin (2\alpha )}$$

Substituting Eq. (14) into Eq. (8) gives

15 $$B = \frac{P\cos (2\alpha )}{4D(1 + v)\sin (2\alpha )}$$

Finally, substituting Eqs. (14) and (15) into Eq. (6), the particular solution for a sectorial plate subjected to a tip concentrated force *P* is given by

16 $$w_{0p} \; = \;\frac{{Pr^{2} }}{4D\sin (2\alpha )}\left( {\frac{\cos (2\alpha )}{(1 + v)}\; + \;\frac{\cos (2\theta )}{(1 - v)}} \right)$$

where the first subscript 0 denotes that this is a particular solution and the second subscript *p* denotes that the deflection is due to an applied tip concentrated force *P*.

### Solution for cantilevered sectorial plates

An eigenfunction expansion is applied to account for the fixed boundary conditions at *r* = *R* for a finite sectorial plate. Noting that the tip concentrated force *P* will cause a symmetric deflection of the plate about the *X*-axis, only the cosine terms of the expansion are needed. Christy et al. (2013) used the eigenfunction expansion for a similar symmetric solution

17 $$w_{j} \left( {r,\theta } \right) = \left( {\frac{r}{R}} \right)^{{n_{j} }} \left[ {\cos (n_{j} \theta )\, + \,b_{j} \cos ((n_{j} - 2)\theta )} \right]$$

This satisfies the free radial edge conditions while allowing adjustment of the particular solution (infinite plate solution) to satisfy the fixed boundary conditions. Each *w*_*j*_ corresponds to a deflection term that contributes to the total deflection, and each *n*_*j*_ is the corresponding eigenvalue for the respective term of the equation. The constants *b*_*j*_ are obtained by imposing the boundary condition *M*_*θ*_(*r*, *α*) = 0 to produce

18 $$b_{j} \; = \;\frac{{n_{j} (1 - v)\cos (n_{j} \alpha )}}{{[4 - n_{j} (1 - v)]\cos ((n_{j} - 2)\alpha )}}$$

Imposing the boundary condition *V*_*θ*_(*r*, *α*) = 0, where *V*_*θ*_(*r*, *α*) is the shear force intensity (N/m units) along a radial edge, gives the characteristic equation for the eigenvalues *n*_*j*_

19 $$n_{j} \left( {1 - v} \right)\sin \left( {n_{j} \alpha } \right) + \left( {4 + \left( {1 - v} \right)\left( {n_{j} - 2} \right)} \right)b_{j} \sin \left( {\left( {n_{j} - 2} \right)\alpha } \right) = 0$$

After the eigenvalues *n*_*j*_ and constants *b*_*j*_ are found, the total deflection equation is given by

20 $$w(r,\theta ) = w_{0} \left( {r,\theta } \right) + \sum\limits_{j = 1}^{\infty } {a_{j} w_{j} \left( {r,\theta } \right)}$$

where *w*_0_ is the particular solution and only the constants *a*_*j*_ remain to be determined by applying the boundary conditions at the fixed support.

Equation (20) indicates that an infinite number of terms are required for the total solution. However, most practical applications will likely require only three or four terms. For example, Christy et al. (2013) and Kennedy et al. (2008) have shown that Carrier and Shaw’s (1950) technique of “averaging” the boundary conditions at the fixed support produces a very accurate total solution using only the first four terms of Eq. (20). The constants *a*_*j*_ are found by applying

21 $$\int\limits_{0}^{\alpha } {w(R,\theta )} {\text{d}}\theta = 0$$

and

22 $$\int\limits_{0}^{\alpha } {\frac{{\partial w\left( {R,\theta } \right)}}{\partial r}{\text{d}}\theta = 0}$$

Equations (21) and (22) permit only two constants *a*_*j*_ to be calculated for two terms in the expansion within the summation of Eq. (20). If more terms (and more constants *a*_*j*_) are desired, then boundary conditions of zero deflection or zero slope can be applied at specific points along the plate’s fixed edge.

### Example

A finite cantilevered sectorial plate, with the geometric and material properties and the loading values given in Table 1, is independently subjected to the three tip loading conditions, as shown in Fig. 1.

**Table 1:** Example constants

*R*	800 mm (31.5 in.)
*α*	0.2 rad (11.44°)
*t*	6.35 mm (0.25 in.)
*E*	70 × 10^3^ MPa (10 × 10^6^ psi)
*ν*	0.33
*P*	4.45 N (1 lb)
*M*_*b*_	110 N mm (1 lb in.)
*M*_*t*_	110 N mm (1 lb in.)

Table 2 displays the three eigenvalues *n*_*j*_ and the corresponding *b*_*j*_ and *a*_*j*_ constants of the eigenfunction expansions for each loading condition for *j* = 1, 2, 3. The tip bending moment and the tip concentrated force solutions both require eigenfunction expansions containing cosines, Eq. (17), because both loadings cause symmetric deflection solutions with respect to the *X*-axis. The tip twisting moment, however, produces an asymmetric solution with respect to the *X*-axis and requires sines in place of the cosines in the eigenfunction expansion (Kennedy et al. 2008). Table 2 also shows that both symmetric solutions produce identical values for the eigenvalues *n*_*j*_ and constants *b*_*j*_; it is only the constants *a*_*j*_ resulting from the particular solutions that are different for the specific loading case. On the other hand, the asymmetric solution corresponding to the twisting moment produces different eigenvalues *n*_*j*_ and constants *b*_*j*_ when compared to the symmetric solutions; further, the first eigenvalue *n*_1_ is now observed to equal unity not zero. For each loading case, Eqs. (21) and (22), along with the specific boundary condition *w*(*R*, *α*) = 0, are applied to produce three constants *a*_*j*_ to be used in the eigenfunction expansion.

**Table 2:** Example coefficients

Loading	*j*	*n*_*j*_	*b*_*j*_	*a*_*j*_
*P*	1	0.000	0.000	9.222 × 10^−2^
	2	1.000	0.201	−1.555 × 10^−1^
	3	7.515	−0.731	−2.796 × 10^−3^
*M*_*b*_	1	0.000	0.000	5.930 × 10^−3^
	2	1.000	0.201	−4.920 × 10^−3^
	3	7.515	−0.731	−8.140 × 10^−5^
*M*_*t*_	1	1.000	−0.201	1.826 × 10^−3^
	2	11.171	−1.753	−7.722 × 10^−5^
	3	13.409	−1.056	1.176 × 10^−5^

Focusing on the total solution for the plate subjected to the tip concentrated force, the constants in the first three rows of Table 2 are substituted into Eq. (17) to provide the first three terms of the eigenfunction expansion, thus

23 $$w_{1p} (r,\theta ) = 1$$

24 $$w_{2p} (r,\theta ) = \left( {\frac{4}{3 + 0.33}} \right)\frac{r}{0.8}\cos \theta$$

25 $$w_{3p} (r,\theta ) = \left( {\frac{r}{0.8}} \right)^{7.515} \left[ {\cos \left( {7.515\theta } \right) - 0.731\cos \left( {5.515\theta } \right)} \right]$$

Finally, the total solution is given by substituting the constants *a*_*j*_ from Table 2, Eqs. (23)–(25), and Eq. (16) with the values from Table 1 into

26 $$w_{p} \left( {r,\theta } \right) = w_{0p} \left( {r,\theta } \right) + a_{1} w_{1p} \left( {r,\theta } \right) + a_{2} w_{2p} \left( {r,\theta } \right) + a_{3} w_{3p} \left( {r,\theta } \right)$$

where the subscript *p* in Eqs. (23)–(26) denotes that the deflection terms are for a plate subjected to a tip concentrated force *P*. It is noted that an analogous procedure is followed for the total solutions for the other loadings.

**Fig. 3:**
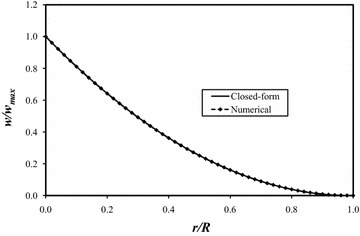
Closed-form and numerical deflections at *θ* = +*α*

The results of the total closed-form solution for the plate under a tip concentrated force were compared to those from a finite element analysis. An ANSYS model using 625 SHELL93 elements was created for this loading; Kennedy et al. (2008) and Christy et al. (2013) describe the ANSYS model in more detail. Figure 3 is an overlay plot comparing the closed-form deflections to the numerical (finite element) deflections along the edge of the plate *θ* = +*α.* Each curve is normalized by dividing it by the maximum closed-form deflection of 2.34231 mm (9.22167 × 10^−2^ in.), which occurs at the tip of the plate. The radial coordinate on the abscissa is normalized by dividing it by the plate’s radius *R*. The two solutions are in near-perfect agreement. Table 3 contains normalized closed-form and numerical deflection values to five significant figures along the edge of the plate *θ* = +*α*; the deflections are normalized by the maximum closed-form deflection.

**Table 3:** Closed-form and numerical deflections at *θ* = +*α*

Normalized position (*r*/*R*)	Normalized closed-form deflection	Normalized numerical deflection
0.0	1.00000 × 10^0^	1.00078 × 10^0^
0.1	8.11252 × 10^−1^	8.11479 × 10^−1^
0.2	6.42049 × 10^−1^	6.41955 × 10^−1^
0.3	4.92393 × 10^−1^	4.92091 × 10^−1^
0.4	3.62288 × 10^−1^	3.61865 × 10^−1^
0.5	2.51757 × 10^−1^	2.51288 × 10^−1^
0.6	1.60864 × 10^−1^	1.60426 × 10^−1^
0.7	8.97613 × 10^−2^	8.94631 × 10^−2^
0.8	3.87709 × 10^−2^	3.87370 × 10^−2^
0.9	8.49806 × 10^−3^	8.84308 × 10^−3^
1.0	−1.23480 × 10^−9^	0.00000 × 10^0^

**Fig. 4:**
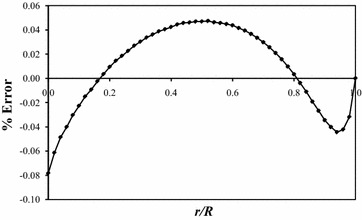
Percent error of plate deflections at *θ* = +*α*

Figure 4 shows the percent error between the two curves plotted in Fig. 3. The percent error is computed by

27 $$\% {\text{Error}} = \frac{{({\text{Closed-form}}\;{\text{deflection}}) - ({\text{Numerical}}\;{\text{deflection}})}}{{{\text{Maximum}}\;{\text{closed-form}}\;{\text{deflection}}}} \times 100\,\%$$

where the difference in the deflections is expressed as a fraction of the maximum closed-form deflection. The maximum error of 0.08 % occurs at the plate’s tip, with the remaining error along the plate’s edge bounded by ±0.05 %.

**Fig. 5:**
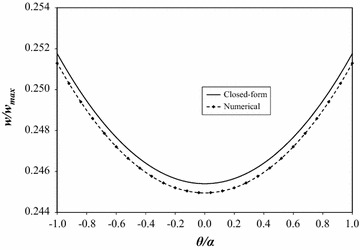
Closed-form and numerical deflections at *r* = *R*/2

**Fig. 6:**
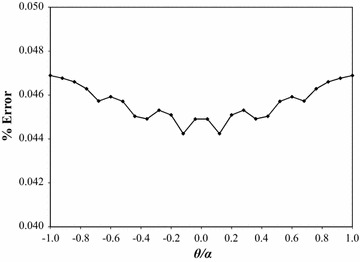
Percent error of plate deflections at *r* = *R*/2

Figure 5 contains plots of the normalized deflections along the arc *r* = *R*/2. The numerical solution is slightly stiffer than the closed-form solution; however, the deflections show similar curvature along the arc traversing from −*α* to +*α*. Table 4 contains normalized closed-form and numerical deflection values to five significant figures along the arc *r* = *R*/2; the deflections in this table are also normalized by the maximum closed-form deflection. Figure 6 shows the percent error of the deflections presented in Fig. 5. The error is symmetric about the plate’s bisector (*θ* = 0) with a maximum error <0.05 %.

**Table 4:** Closed-form and numerical deflections at *r* = *R*/2

Normalized position (*θ/α*)	Normalized closed-form deflection	Normalized numerical deflection
1.00	2.51757 × 10^−1^	2.51288 × 10^−1^
0.92	2.50769 × 10^−1^	2.50302 × 10^−1^
0.84	2.49868 × 10^−1^	2.49402 × 10^−1^
0.76	2.49051 × 10^−1^	2.48588 × 10^−1^
0.68	2.48319 × 10^−1^	2.47862 × 10^−1^
0.60	2.47670 × 10^−1^	2.47211 × 10^−1^
0.52	2.47104 × 10^−1^	2.46647 × 10^−1^
0.44	2.46620 × 10^−1^	2.46170 × 10^−1^
0.36	2.46218 × 10^−1^	2.45769 × 10^−1^
0.28	2.45897 × 10^−1^	2.45443 × 10^−1^
0.20	2.45656 × 10^−1^	2.45205 × 10^−1^
0.12	2.45496 × 10^−1^	2.45053 × 10^−1^
0.04	2.45415 × 10^−1^	2.44966 × 10^−1^
−0.04	2.45415 × 10^−1^	2.44966 × 10^−1^
−0.12	2.45496 × 10^−1^	2.45053 × 10^−1^
−0.20	2.45656 × 10^−1^	2.45205 × 10^−1^
−0.28	2.45897 × 10^−1^	2.45443 × 10^−1^
−0.36	2.46218 × 10^−1^	2.45769 × 10^−1^
−0.44	2.46620 × 10^−1^	2.46170 × 10^−1^
−0.52	2.47104 × 10^−1^	2.46647 × 10^−1^
−0.60	2.47670 × 10^−1^	2.47211 × 10^−1^
−0.68	2.48319 × 10^−1^	2.47862 × 10^−1^
−0.76	2.49051 × 10^−1^	2.48588 × 10^−1^
−0.84	2.49868 × 10^−1^	2.49402 × 10^−1^
−0.92	2.50769 × 10^−1^	2.50302 × 10^−1^
−1.00	2.51757 × 10^−1^	2.51288 × 10^−1^

**Fig. 7:**
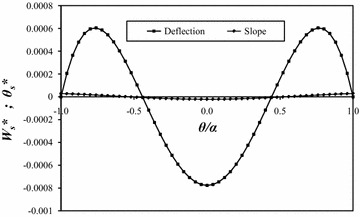
Closed-form deflection and slope along the fixed circumferential support *r* = *R*

Figure 7 contains plots of the resulting closed-form deflection and slope along the fixed boundary. The normalized support deflection *W*_s_* is obtained by dividing the support deflection by the maximum closed-form deflection *w*_*max*_. The normalized slope at the support *θ*_s_* is obtained by dividing the support slope by the ratio of the maximum closed-form deflection to the plate’s radial length *w*_*max*_/*R*. The true boundary conditions are not satisfied exactly, as the curves are observed to oscillate about the exact zero deflection and slope boundary conditions. However, these deviations from zero are very small, indicating that the “averaged” boundary conditions [Eqs. (21) and (22)] provide a sufficiently accurate solution for most practical applications. Finally, it is observed that the boundary condition *w*(*R*, *α*) = 0, applied at a single point, ensures that the deflection is identically zero at *θ* = +*α* and −*α*.

## Conclusions

A closed-form solution for a finite cantilevered sectorial plate subjected to a tip concentrated force is presented. Since the particular solution was not found in the literature, it is derived in this paper. A symmetric eigenfunction expansion is used to augment the particular solution to account for the fixed boundary conditions at the circumferential support. Deflections from the total closed-from solution are found to be in excellent agreement with deflection results from a finite element analysis; the error is always within 0.08 % for the given example. Finally, the total closed-form solutions for a cantilevered sectorial plate subjected to independent applications of a tip concentrated force, a tip bending moment, and a tip twisting moment, are compiled.
